# Achieving universal health coverage through voluntary insurance: what can we learn from the experience of Lao PDR?

**DOI:** 10.1186/1472-6963-13-521

**Published:** 2013-12-17

**Authors:** Sarah Alkenbrack, Bart Jacobs, Magnus Lindelow

**Affiliations:** 1 Futures Group, Washington, DC, USA; 2 Social Health Protection Programme (SHPP), Deutsche Gesellschaft für Internationale Zusammenarbeit, Phnom Penh, Cambodia; 3 Human Development Department, The World Bank, Brasilia, Brazil

**Keywords:** Health insurance, Community based health insurance, Risk-protection, Health financing, Lao PDR, South East Asia, Voluntary health insurance, Universal health coverage, Enrolment

## Abstract

**Background:**

The Government of Lao Peoples’ Democratic Republic (Lao PDR) has embarked on a path to achieve universal health coverage (UHC) through implementation of four risk-protection schemes. One of these schemes is community-based health insurance (CBHI) – a voluntary scheme that targets roughly half the population. However, after 12 years of implementation, coverage through CBHI remains very low. Increasing coverage of the scheme would require expansion to households in both villages where CBHI is currently operating, and new geographic areas. In this study we explore the prospects of both types of expansion by examining household and district level data.

**Methods:**

Using a household survey based on a case-comparison design of 3000 households, we examine the determinants of enrolment at the household level in areas where the scheme is currently operating. We model the determinants of enrolment using a probit model and predicted probabilities. Findings from focus group discussions are used to explain the quantitative findings. To examine the prospects for geographic scale-up, we use secondary data to compare characteristics of districts with and without insurance, using a combination of univariate and multivariate analyses. The multivariate analysis is a probit model, which models the factors associated with roll-out of CBHI to the districts.

**Results:**

The household findings show that enrolment is concentrated among the better off and that adverse selection is present in the scheme. The district level findings show that to date, the scheme has been implemented in the most affluent areas, in closest proximity to the district hospitals, and in areas where quality of care is relatively good.

**Conclusions:**

The household-level findings indicate that the scheme suffers from poor risk-pooling, which threatens financial sustainability. The district-level findings call into question whether or not the Government of Laos can successfully expand to more remote, less affluent districts, with lower population density. We discuss the policy implications of the findings and specifically address whether CBHI can serve as a foundation for a national scheme, while exploring alternative approaches to reaching the informal sector in Laos and other countries attempting to achieve UHC.

## Background

Since the World Health Organization (WHO) endorsed a resolution encouraging countries to progress towards universal health coverage (UHC) [1], much momentum has been gained and the world is in the midst of a “financing transition”. This transition refers to a movement away from financing health care through out-of-pocket payments towards health insurance and risk-pooling schemes, to ensure that the entire population has affordable access to key health interventions without risk of impoverishment [2]. In December 2012, the United Nations passed a resolution urging member states to provide affordable health care for all, thereby increasing government accountability for pursuing the goal of universal coverage.

Many high-income countries that are progressing towards, or have already achieved, universal coverage have relied heavily on general taxation, social health insurance or a mix of both [3,4]. However, in many low- and middle income countries striving towards universal coverage, a pluralistic health financing system typically evolves whereby a mix of health insurance and risk-protection schemes are targeted at distinct socio-economic groups. The two most common schemes are social health insurance for formal sector workers and general tax finance for the poor and vulnerable, as this latter group is generally accepted as the responsibility of the government. The non-poor informal sector, which generally comprises a substantial share of the population in low- and middle-income countries, is much more difficult to reach and is often the last to be covered. This is because these workers are often difficult to identify, do not have formal employer-employee relationships that are conducive to collecting contributions, and may have irregular incomes that lead to defaults on contributions.

In an effort to extend coverage to the informal sector, many countries have implemented community-based health insurance (CBHI), or some other model of voluntary health insurance (VHI). However, a large body of literature shows that the vast majority of CBHI and other voluntary schemes fail to reach a large proportion of their target population, and in the absence of subsidies most schemes exclude the poor [5-9]. Despite the challenges of expanding coverage through CBHI and other voluntary health insurance schemes, these schemes continue to feature prominently in low-income countries’ health financing strategies. Reasons for relying on CBHI relate to the desire to bypass national-level challenges such as national tax collection systems or extension of social health insurance from the formal sector to the often poorer informal sector population [10]. Some are optimistic that CBHI will serve as a stepping stone to national insurance schemes, and cite historic precedents, such as Japan, where voluntary insurance schemes evolved into nationwide health insurance schemes [11].

The situation in Lao PDR is not dissimilar to other countries that have relied, at least to some extent, on voluntary health insurance to cover the informal sector. In Laos, CBHI has been operating since 2001 with technical and financial assistance from various donors. However, following 12 years of operation, the scheme covers just over two percent of the population [12]. Nevertheless, the government’s health financing strategy and support by some development partners is premised on the belief that the scheme can be expanded and that CBHI can provide an important basis for making progress towards universal coverage. But given CBHI’s low coverage to date, it is worth understanding both the factors affecting enrolment in voluntary health insurance and the prospects of expanding CBHI to new districts in the future. Thus, the objectives of this study are twofold: 1) to examine the relative importance of factors driving *household* enrolment in areas where CBHI is *already operating*, with a particular interest in knowing whether health status and socioeconomic status are significant determinants of enrolment; 2) to explore the likelihood that CBHI can be further expanded *geographically* to *new districts* by comparing characteristics of districts with and without CBHI. Given the variation in CBHI schemes globally, these policy questions relate not just to CBHI schemes, but to voluntary health insurance more broadly. Thus, the results from the study are used to draw inferences about the role that voluntary insurance can play in achieving universal coverage in Lao PDR, as well as other low- and middle-income countries.

### The setting

Lao PDR remains one of the poorest countries in South-East Asia, and is ranked 138th out of 187 countries on the Human Development Index [13]. The population of 6.1 million is low by regional standards and, with 49 distinctive ethnic groups and four main ethno-linguistic families, is the most ethnically and linguistically diverse in mainland South-East Asia [14]. Although the country has achieved much progress in reducing poverty and child mortality over the last decade, poverty rates are high and significant challenges remain in improving health outcomes, which are among the poorest in the region. Like many of its neighbours, Laos is transitioning from an agricultural socialist economy to a market-oriented economy, and growth is relatively strong due to increased integration with neighbouring countries and development of mining and hydropower [15]. Growth in these sectors is expected to increase fiscal space, which is currently low by regional standards [16].

### Health care organization and financing

The main network for the provision of health services is the public sector but the shift to a market-economy has facilitated growth of the private health sector, which is still relatively small, largely unregulated, and exists mainly in urban areas. Utilization of government services is low: the likelihood of an individual seeking care from a modern health provider when ill was 18% in 2008 [17]. In urban areas, private pharmacies and clinics are often the first choice of care [18,19] and many people use health services in neighbouring Thailand provinces, where the majority of health workers speak Isan Thai, a dialect of the Lao language, as their native tongue [20,21].

Although historically, health care was financed through the government budget, revolving drug funds and user fees were adopted in the 1990s to secure access to essential medicines and increase revenues to the health sector [22,23]. In 1995, a user fee exemption policy was put in place, but was not operationalized due to lack of clear criteria for identifying the poor, and lack of financial support to providers for exempted patients [24]. Thus, the burden of financing falls largely on households, with out-of-pocket payments accounting for 61% of overall health spending in 2010 [25]. Government spending on health as a percentage of total health expenditures is much lower than other countries in the region (33% compared with 75% in Thailand) [25] and accounts for less than 1% of GDP [17]. Much of the public expenditure on health (75%) covers salaries for health care workers, and therefore facilities are dependent on revenues from the revolving drug funds to cover recurrent costs and to fund top-ups to staff to augment their low salaries [26]. Thus, there is an incentive for providers to overprescribe non-essential and non-generic drugs and diagnostic tests. Drugs are often charged at a higher profit margin than the official rates and charges are not always displayed in pharmacies [21,27].

**Figure 1:**
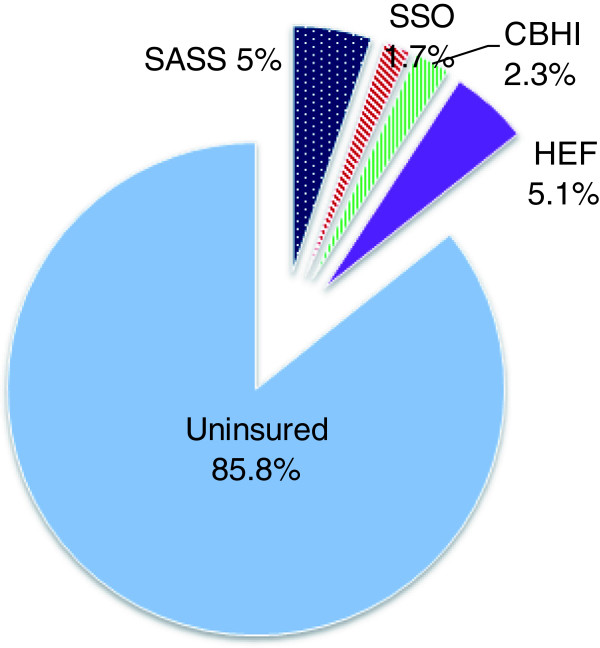
Coverage of health insurance and health equity funds in Lao PDR, 2011.

In an attempt to increase access to health services, increase financial protection, and generate resources for the health sector, the Government of Laos is trying to expand coverage of health insurance and risk protection schemes. The ultimate goal is achievement of universal coverage [28], and to this end the government has introduced four main schemes: a mandatory Civil Servants’ Scheme for government employees (now termed State Authority for Social Security (SASS)); a mandatory Social Health Insurance scheme for private and state-owned enterprises, run by the Social Security Organization (SSO); voluntary community-based health insurance for the informal sector and self-employed workers; and health equity funds (HEFs) for households living in extreme poverty. However, outside the SASS scheme, which targets approximately 5.2% of the population and reaches approximately 98% of its target group, coverage of schemes is low, as Figure 1 shows. Community-based health insurance targets roughly 50% of the population, but covers only 2.2% of the population; SHI targets the formal sector, which comprises roughly 5% of the population, but the scheme covers only 1.7% of the population; and HEFs target the poor – roughly one third of the population – but cover approximately 5% of the population. Thus, only about 14% of the population is currently covered by risk-pooling schemes.

### Overview of CBHI

The Lao CBHI scheme bears closer resemblance to the voluntary schemes in Asia e.g., voluntary health insurance in Vietnam and China, than to other CBHI schemes in sub-Saharan Africa, in that it is predominantly managed by the government rather than the community. Risk-pooling takes place at the district level whereby the MOH contracts with district hospitals to provide services for CBHI members and a gatekeeping system requires members to first seek services at the contracted facility in their district before being referred to central or provincial hospitals. In provincial capitals where CBHI is operational, however, members can directly access provincial or regional hospitals without referral. The benefit package for members covers outpatient and inpatient services and drugs purchased at hospitals and is similar to the health care benefits in the country’s two formal sector schemes.

The main target group for the CBHI scheme is defined as households who are self-employed or working in the informal sector and are not covered by other social health protection schemes. Enrollment takes place at the household level and the premium varies according to urban or rural residence and number of household members. The contribution rates were originally set at between 2.5 to 3 percent of average household income [29] for the country, and therefore would account for a greater share of income among the informal sector. Scheme contributions are collected on a monthly basis by village collectors. These collectors are mostly appointed by village authorities. They receive very small fees for each enrolled household, often incur uncovered costs such as fuel, and often have little motivation to perform the expected tasks. As a result of problems with the fee collection system, e.g., village collectors do not always collect contributions, some villages have moved to a system whereby villagers make their payments directly to the CBHI account manager at the district hospital.

Implementation of CBHI in Laos has been highly selective. Predominantly urban and semi-urban areas were targeted first, because health care services were perceived to be of a reasonable quality in those areas and the socioeconomic status of the target population was deemed high enough to make the premiums affordable. The targeted villages were also selected for their close proximity to the district hospital. The rationale for this selective implementation was to strengthen the scheme in the areas that were considered the “easiest” to reach. However, by December 2011, the schemes were operating in 26 districts, where they reached only 8% of the population in those districts (and 11% of the villages that had been targeted in those districts). The Government of Laos plans to expand to more remote areas, where enrolment is expected to be even more challenging than in urban areas. However, evidence about the profile of districts that have been both targeted and not targeted by CBHI has been largely anecdotal. More information is needed about why household enrolment is so low in the targeted districts, but it is also important to understand district-level factors that would hinder or facilitate future expansion.

This study explores the determinants of enrolment at the household level through use of quantitative and qualitative methods, and then goes one step further to systematically assess how districts with and without CBHI differ, in terms of distance to health facilities, ethnicity (a proxy for health-seeking behavior), population density, and other factors that are expected to affect the demand for health care and insurance. The objective is to use the findings from the household and district level to shed light on the prospects for expanding CBHI nationally.

## Methods

In this study we examined the prospects of expanding enrolment of CBHI using two distinct approaches. To examine the determinants of enrolment in CBHI at the household level, we collected data using a household survey and focus group discussions. To examine the factors associated with roll-out of insurance at the district level, we compiled a database of district level variables and compared characteristics between districts with and without CBHI. These approaches are discussed in detail, below.

### Household and village surveys

The household survey was conducted using a case-comparison study design, with households enrolled in CBHI (herein referred to as CBHI households) and unenrolled households recruited from villages where CBHI had been implemented. The sample consisted of 3000 households, selected from 87 villages across 6 districts (3 provinces: Hatxaifong and Sissatanak in Vientiane Capital; Viengkham, Phonehong, and Keodoum in Vientiane Province; and Champasack district in Champasak province). CBHI households were eligible for the study if they had been enrolled for at least one year. This one year period was defined because the recall period for health care utilization and expenditures was one year. A two-stage cluster sample was randomly selected: first, villages were selected with probability proportional to population; then households were randomly selected in one of two ways. CBHI member households were randomly selected from member lists in villages, while comparison households were randomly selected from the village registry. For every CBHI household, two comparison households were selected. The rationale for this ratio was to ensure an adequate pool of comparison households was available for the impact evaluation, the results of which are presented separately [30]. The sample of CBHI households comprised 30% of all households enrolled in CBHI across the six districts at the time of the study.

Data collection took place from February to April, 2009. The household survey included multiple measures of health status and risk preferences as well as factors related to preferences for modern health care. We also measured perceived quality of care at the household and village level. To measure socioeconomic status we developed an aggregate consumption measure, by compiling data on the types of food and non-food items that households either purchased, produced themselves, or obtained through nonfinancial transactions. Data were then combined to give per capita consumption quintiles, with per capita rates calculated using an equivalent scale.

Prior to conducting the survey, interviewers obtained informed consent from participants and screened households to ensure eligibility. Surveys were administered to the head of household, where possible. The interviewee was also asked to answer questions about other household members. The response rates for the CBHI and non-CBHI strata were 99.7% and 96.9%, respectively.

Descriptive data analysis was performed using Stata 10.1 to better understand which types of households typically enrol in CBHI. CBHI and non-CBHI households were compared on a range of characteristics and univariate analysis was performed. All estimates account for sampling weights and village-level clustering. To perform the multivariate analysis on the determinants of enrolment, a probit model was used. The probability of enrolment is a function of individual/household and village characteristics, such that:

$$\text{Pr}\left(y=1|x\right)=f\left(X_{1,}X_{2,}\epsilon \right)$$

where y = 1 for an enrolled household and y = 0 for an uninsured household; *X*_*1*_ represents individual and household characteristics; *X*_*2*_ is a vector of village level characteristics; and ϵ represents the error term. The model uses sampling weights and accounts for the cluster effects at the village level. However, because the probit model assumes a nonlinear relationship between the independent variables and the outcome variable, the marginal effects are not constant. Instead, the relationship between an x-variable and the outcome depends on the level of x as well as the level of other independent variables. Therefore, to better understand the relationship between independent variables and the outcome (enrolment), predicted probabilities were estimated. These predicted probabilities represent the probability that a household with a certain characteristic will enrol in CBHI when all other factors are held constant at their mean value and were estimated using the *SPost* programme in Stata [31]. Comparing predicted probabilities for “representative individuals” shows how the probability of enrolment changes as the variable of interest changes.

Because enrolment was measured using a cross-sectional survey, it was important to acknowledge that the relationship between independent variables and enrolment could be endogenous and endogeneity of consumption was a primary concern. To partially address this endogeneity problem, health care expenditures were excluded from the aggregate consumption measure and quintiles of consumption were used in place of an absolute measure. However, it is still possible that the relationship between consumption and enrolment is endogenous (i.e., the level of non-medical consumption in a household could still be affected by whether or not the household took up CBHI).

In addition to using consumption quintiles to look at the relationship between wealth and enrolment, a household asset index, constructed using principal components analysis, was used in the multivariate analysis. Relative to consumption, an asset index is more likely to reflect longer-run household wealth or living standards, and is less likely to account for short-run interruptions or shocks to the household [32]. Although there is some correlation between consumption and the asset index, the asset index also has an independent effect on enrolment, which justifies its inclusion in the model.

Despite the steps taken to reduce endogeneity, the main limitation of this study is the cross-sectional nature of the data, which makes it difficult to infer causation between an independent variable and enrolment. However, qualitative work conducted with CBHI members and non-members as part of this study, help to validate and support interpretation of results. Detailed findings from focus group discussions are presented separately [30], but are briefly described below and referenced in this paper where appropriate.

### Focus group discussions with households

Focus group discussions (FGDs) were held in six villages where CBHI is running: three groups included members and three included non-members, which totalled 55 individual participants. Villages were purposively selected from the list of villages where the survey was conducted previously. An effort was made to choose villages with a range of CBHI coverage levels, varying distance to the district hospital, and of varying size. The FGDs focused on five main topics: knowledge of CBHI (and details of implementation); motivation for enrolment/non-enrolment/dropping-out; experiences and perceptions of the scheme and of the health care system; impact of enrolment/non-enrolment on use of services, expenditures, and source of care; and recommendations for improving the scheme. The FGD guide was translated into Lao and discussions were conducted using a local female moderator with experience conducting FGDs in Laos. Training and pilot testing were conducted over a four day period in Vientiane Capital in May 2009, and data collection took place thereafter and lasted for 6 days. Verbal consent was given by all participants and all discussions were audio recorded. Data were translated and transcribed and analysed using thematic analysis [33] and were used to help interpret and validate the quantitative findings.

### District level secondary data

In the second phase of the study, we examined the likelihood of expanding enrolment to new areas. To do this, we compiled district-level data and compared characteristics of districts with and without insurance to assess the likelihood of scaling-up geographically. We use a combination of univariate and multivariate analyses to explore differences between the two groups. The multivariate analysis is a probit model, which models the factors associated with roll-out of CBHI to the districts. The independent variables include: poverty rate, population density, literacy rate, the percentage of nonagricultural employment, the percent of households with electricity, distance to the district hospital, ethnicity (i.e., Tai-Kadai vs. other), and religion (i.e., Buddhist vs. other). These indicators were compiled from the Lao Socioeconomic Atlas [14] and data from the MOH, CBHI office.

### Ethics

The MoH approved the implementation of the survey and focus group discussions and ethical approval for the study was granted by the ethics committees at the National Institute of Public Health in Laos and London School of Hygiene and Tropical Medicine (LSHTM) in the United Kingdom.

## Results

Table 1 presents the results of the descriptive statistics at the household level. The results show that CBHI households are larger, more likely to be married, and more educated than uninsured households. There is no difference in ethnicity between CBHI and comparison households: most households in the sample belong to the Tai-Kadai ethnic group. CBHI households have significantly higher consumption levels than uninsured households but similar *per capita* consumption levels. Per capita income levels are also similar between groups and are just below the national average of $880 per capita. There is no significant difference in the number of CBHI and comparison households living below the national poverty line: across the sample, 21.4% of households live in poverty. This percentage is less than the estimated poverty headcount in the population (37%). Thus, the sample has a lower average income level than the population but also has fewer households living in poverty, which makes sense given that CBHI targets the near-poor informal sector, but not the poorest or richest households. Among households in which the household head is employed, the insured are more likely than the uninsured to hold a long-term contract.

**Table 1:** Background characteristics of CBHI and comparison populations

	**CBHI (n = 1000)**	**Comparison (n = 2000)**	**p-value**
**Sociodemographic characteristics**			
Mean household size (persons)	5.3	4.7	<0.001**
Marital status of household head (% married)	84.2%	80.4%	0.027*
***Education***			
Highest level = any primary	43.1%	42.7%	0.866
Highest level = any secondary	31.6%	37.2%	0.028*
Highest level = university/institute	5.1%	2.3%	0.002**
Highest level = vocational	11.8%	8.4%	0.020*
Age of HH head (mean years)	52.4	48.4	<0.001**
HH is member of ethnic majority (1 = Tai-Kadai; 0 = other)	98.6%	98.2%	0.404
Total annual household consumption ($US)	$3,162	$2,729	<0.001**
Total annual per capita consumption, mean ($US)^a^	$754	$741	0.531
Total annual per capita income, mean ($US)	$863	$845.9	0.822
HHs living below $1.25 per day	21.6%	20.3%	0.435
***Employment status***			
Not working for money	21.1%	17.2%	0.009**
Family farm-based agriculture	24.0%	22.8%	0.644
Small-scale trading or family business	26.4%	31.2%	0.039*
Work for someone else	28.5%	28.8%	0.878
HH heads with long-term employment contract (12 months +)	17.2%	11.6%	0.002**
Household is located in urban area (vs. semi-urban or rural)	30.1%	33.9%	0.413
**Health status and risk aversion**			
HHs in which avg self-rated health is <3 on scale of 1 to 5	19.4%	14.9%	0.023*
HHs in which someone has disability or chronic condition	23.4%	14.5%	<0.001**
HHs in which someone had difficulty with activities in 3 months	16.3%	11.0%	0.008**
HHs in which s/o experienced deterioration of health in past year	11.9%	8.5%	.034*
Risk preferences: head of household is risk-averse^b^	37.1%	41.6%	0.041*
**Other risk variables**			
HHs with any member age 65+	28.0%	21.9%	.001**
HHs with any member age 0-5	37.0%	37.6%	0.754
Mean # of females 15–49	1.6	1.4	<0.001**
HHs in which a woman has given birth in past 2 years	15.7%	13.9%	0.261
HHs with a pregnant woman	4.4%	2.3%	.004**
**Attitudes towards sources of care and quality perceptions**			
HH respondents recommending a government hospital for an uninsured friend.			
A severe condition/emergency?	99.4%	99.5%	0.669
A moderate condition?	94.6%	92.6%	0.138
A minor condition?	97.5%	96.8%	0.42
HH respondents stating that services at district hospital are good	75.4%	64.8%	<0.001**
**Exposure to CBHI and trust in scheme**			
HH attended CBHI campaign	92.5%	66.2%	<0.001**
How many of your close relatives/friends had joined CBHI prior to enrolment? (or how many are enrolled now?)			
None	4.5%	30.7%	
Some	49.2%	48.9%	<0.001**
Many	46.3%	20.4%	
HHs reporting trust that contributions will be used properly	92.5%	69.7%	<0.001**
HHs reporting that members will get the benefits they pay for when they need them	95.8%	69.4%	<0.001**

*Significant at 5%;**significant at 1%. Reported results are based on t-tests of means for continuous variables and chi-squares for proportions/categorical variables. All estimates account for sampling weights and village-level clustering. ^a^Per capita expenditure was calculated using adult equivalents. ^b^This question presents the respondent with a gamble in which he/she must guess which hand contains money. The first pick is the same regardless of the hand selected but in the next bets, the stakes become increasingly higher. The variable was dichotomized to differentiate those who were completely risk averse from those who will take at least some risk. This methodology was adapted from a study in India by [34] and was also used by Krishnan et al, 2010 [35].

In terms of health status, CBHI households are less healthy than uninsured households, as shown by the CBHI group’s poorer self-rated health, higher prevalence of disability or chronic illness, higher proportion of households who reported difficulty performing activities, and a higher proportion of households in which a household member has deteriorating health. CBHI households also have more elderly household members, more women of reproductive age, more pregnant women, and are relatively less risk-averse than uninsured households.

Attitudes towards different sources of care serve as proxies for preferences for modern health care over traditional. The descriptive findings show that attitudes are similar among CBHI and non-CBHI households. However, CBHI households report a higher perception of quality of health care at the district hospital. CBHI members are also more likely than the uninsured to have attended a CBHI campaign, to have more close relatives and friends in the scheme, and to place higher trust in the scheme.

### Multivariate findings

**Figure 2:**
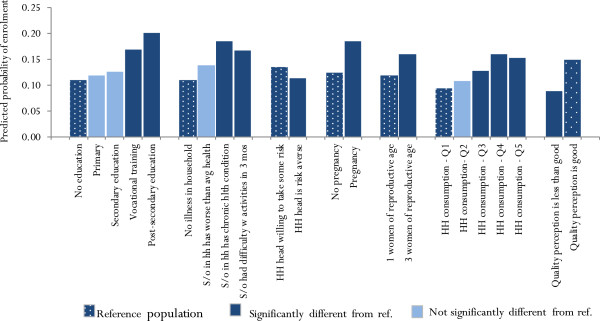
Predicted probability of enrollment by household characteristics.

Figure 2 shows the predicted probabilities of enrolment for representative households. Households with primary or secondary education have a higher predicted probability of enrolment than those with no education, but this difference is not significant. However, the probability of enrolment is significantly higher for those with vocational training or post-secondary education.

Health status and economic well-being, and their relationship with enrolment in CBHI, were of particular interest in this study. Results show that households in which a family member has either a chronic illness or had difficulty performing regular activities in the past three months (a proxy measure for illness) were significantly more likely to enroll in CBHI than households with no signs of illness. Although CBHI members had higher self-assessed health than non-members, this relationship was not significant. A pregnancy in the household and a higher number of women of reproductive age in the household was significantly associated with enrolment, but having an elderly family member or a child under the age of five was not significantly associated with enrolment.

The FGDs support the quantitative findings: in all FGDs, the most frequently cited reason for enrolling was that family members suffer from chronic conditions, while in non-member households the second most frequently reported reason for *never* enrolling in the scheme was that people were healthy. Two examples from the FGDs illustrate these points.

“Joining CBHI is really convenient as I often see doctors about my diabetes and high blood pressure. Sometimes it is not only me who sees the doctors, but also my family members: one person has a fever or another person has a problem with his stomach”.

-CBHI member from Ban Donetalarth, Champasak district

“No one in my family gets sick; I am not really interested in this scheme. When someone in my family gets sick, we just go to the hospital like normal patients”.

-Non-member from Ban Chompettai, Sissattanak district

The study found that people who are very risk averse are actually *less* likely to enroll in CBHI. Qualitative interviews shed light on why this may be the case. Although the majority of the respondents in the FGDs reported that enrolling in CBHI allows people to minimise their risk, some felt that enrolling in CBHI is a risky venture and that enrolment actually *increases* risk, because one can’t be sure that benefits will be delivered when they are needed.

“I am taking a risk by putting money into the scheme because after two years if the scheme collapses I will not get anything from it.”

-Member from Ban Thamouong, Hatxaifong district

When other factors are held constant, households that are better-off financially (measured by per capita consumption) are significantly more likely to enroll in CBHI. However, the marginal effect of consumption on enrollment diminishes as wealth increases. Households in the highest quintile are not much more likely to enroll in health insurance than those in the fourth quintile. This makes sense given that CBHI does not target the rich. According to a FGD participant, *“rich people are not interested in joining this scheme because they have money and when they get sick they can choose wherever they like to go for treatment; mostly they will go to private clinics, then to the central hospital or to Thailand”.* The most frequent reason for never enrolling in CBHI was the inability to afford the premiums. A FGD participant explained why his family’s financial situation prevents him from enrolling:

“For me, it’s already difficult to pay for our monthly expenditure. I don’t have enough money. My four children also go to school; sometimes I cannot earn money for their school on time. Then I have other expenses such as water bill, electricity bill, and rice farming. It costs more than 1 million kip per year. So the money I earn is spent on many things. There are eight people in my family; they sometimes get sick but not very serious illness. I sometimes borrow money from others when my children get sick”.

-Non-member from Ban Phonehang, Viengkham district

The study found that almost all households in the sample belong to the Tai-Kadai ethnic group, and almost all households have a preference for modern health care over traditional care. Therefore, these factors do not explain differences in enrollment in this sample. As shown in Figure 2, a higher perception of quality of health care is associated with enrollment in CBHI. However, this relationship could be endogenous, due to the cross-sectional nature of the data: it is plausible that higher quality reported by CBHI members is a *result* of being in the scheme, rather than a factor affecting enrolment (e.g., CBHI members may have more contact with the district hospitals or a better experience overall because they don’t have to pay for health care). The focus group discussions shed light on the relationship between quality perceptions and enrolment, as several dimensions of quality were explored in the qualitative work (see Alkenbrack, 2011). Across all six FGDs, CBHI members and non-members agreed that non-members, who pay for services at the point of service delivery, receive services faster than CBHI members regardless of the urgency of health care needs.

“When CBHI members go to the hospital and show their membership card, the hospital workers ignore them and make them wait for a very long time. But if people with money come to the hospital, the staff gives them faster services — they need not wait long! In some cases, when CBHI members have an emergency or very serious illness, they do not receive priority treatment. Instead, they are kept for a very long time and have to wait their turn. So some people would die before they get their turn! This is why some people drop out”.

-Non-member from Ban Thamouong, Hatxaifong district

Across all six villages where FGDs were held, participants complained that CBHI members usually receive low quality drugs, while non-members are prescribed a variety of more expensive drugs. CBHI members are also reportedly treated with less respect than cash-paying patients. Aside from the differential treatment given to members and non-members, health care workers reportedly provide faster treatment to patients who have money or who have relatives working in the hospital. Several participants also mentioned that children are given preferential treatment over adults. However, quality of service delivery is reportedly poor in general. Both members and non-members reported that health care staff members do not have the skills to diagnose health problems and that productivity is low. For example, some FGD participants described the behaviour of health care workers as *“lazy”,* explaining that *“they just sit there and ignore patients with CBHI”.* Although a few respondents complained about the cleanliness of the hospital, most participants felt that the facilities were clean but that lack of equipment is a problem in the district hospitals. Thus, results from the FGDs contradict the quantitative findings, which indicated that CBHI members have higher perceptions of quality than non-CBHI members. It is therefore more likely that members who have positive experiences with CBHI *maintain* enrolment in CBHI, and less likely that perceptions of good quality of care at district hospitals are enticing households to enrol in the scheme.

### District-level findings (from secondary data analysis)

The district-level findings compare CBHI and non-CBHI districts on various characteristics (See Table 2). Findings from the univariate analysis show that, relative to non-CBHI districts, CBHI districts have a significantly higher population density, lower poverty rates, higher literacy rates, and a higher proportion of the population working in the non-agricultural sector. CBHI districts are also significantly more likely to have electricity than non-CBHI districts. On average, residents of non-CBHI districts are located three times further from a health facility than CBHI districts, and are significantly more likely to belong to an ethnic minority and a non-Buddhist households. Results from the multivariate analysis (probit), are shown in Table 3 and show that only two variables are significantly associated with enrolment in CBHI, including a higher percentage of labor in non-agricultural activities and closer proximity to the nearest health facility.

**Table 2:** Univariate analysis of differences between CBHI and non-CBHI districts

	**CBHI (26)**	**Non-CBHI (114)**	**t-test**
Population density (per km2)	184.62	46.42	-2.571*
Poverty rate (%)	29.6	40.1	3.386**
Literacy rate (%)	79.00	64.2	-4.061**
% of labor non-agriculture	33.38	12.09	-5.868**
% HHs with electricity	73.7	41.87	-5.948**
Avg time to nearest health center (mins)	57.65	161	4.5322**
% pop Lao-tai (vs. ethnic minority)	56.09	26.93	-3.741**
% pop Buddhist	79.48	53.29	-3.71**

*Significant at 5%;**significant at 1%. Reported results are based on t-tests of means. When these variables are modelled using a probit model, only two variables are significant: the percentage of labor in non-agriculture activities, and time to the nearest health facility.

**Table 3:** Marginal effects of district-level factors associated with roll-out of CBHI

**CBHI**	**M.E.**	**S.E.**	**P > |z|**
Poverty rate (%)	0.120446	0.261431	0.636
Literacy rate (%)	-0.0871	0.287819	0.759
Population density (per km2)	-0.00048	0.000339	0.121
% of labor non-agriculture	0.004238	0.002691	0.038*
% HHs with electricity	0.002023	0.001606	0.132
time to nearest health center (mins)	-0.00152	0.000426	0.011*
% pop Lao-tai (vs. ethnic minority)	0.00101	0.000915	0.214
% pop Buddhist	-0.00185	0.001206	0.136
*Number of observations*	*137*		

*Significant at 5%; Pseudo R2 = 0.3671; Log pseudoliklihood = -41.195; Prob > chi2 < .001.

## Discussion

The findings generated from this study reveal useful information about CBHI in Laos, its members, and their experiences and allow us to gain insight into the prospects of further expansion of CBHI in Laos. Our first research question examined the relative importance of factors driving households’ enrolment in CBHI and found that the decision to enroll in CBHI is influenced by a range of factors at the household level including education, health status, risk attitudes, socioeconomic status, and quality perceptions. Four of the household level findings are of particular interest. First, the study found that illness is driving enrolment in CBHI, indicating that the scheme suffers from adverse selection, and the use of multiple health status measures through the household surveys, as well as the qualitative work, help to understand this relationship. The finding that adverse selection is present in the scheme supports literature from Senegal, Thailand, India and China [36-40] but is inconsistent with results from other studies that did not find a link between health status and enrolment [41-45]. From a public health perspective the link between poor health and higher enrolment is encouraging, as it indicates that households with the greatest need for health care services are purchasing insurance. However, from a sustainability perspective, the results are discouraging. Given that all members play a flat-rate premium regardless of their risk profile, adverse selection can drive up the cost of health care per insured member and can ultimately threaten the sustainability and financial viability of the scheme.

Another key finding is that the poor are less likely to enroll in health insurance than the better-off, which raises concern in that the scheme does not provide financial protection for the poorest. These results are consistent with much of the enrolment literature [37,40,41,43,46,47]. With the exception of a few villages where health equity funds are operating alongside CBHI schemes, there are no subsidies in place to cover the cost of CBHI premiums for the poor, and therefore no cross-subsidization in overall health care financing, as another study from Laos confirms [48]. Nor is a systematic targeting scheme in place for identifying poor households. Given that the poor are most vulnerable to catastrophic health payments [49,50], it is important that strategies to expand risk-protection schemes target the poorest. However, given the current financing arrangements in Laos, CBHI is unlikely to increase financial protection among the poor in areas where subsidies for the poor are not available.

The third notable finding from the household survey is that households with greater risk aversion are less likely to enrol in insurance. On the face of it, this finding seems to contradict insurance theory, which holds that individuals who are relatively more risk averse will be more likely to enrol in health insurance due to the desire to protect themselves from health-related financial loss in the future [51]. However, it is important to note that expected utility theory assumes that insurance is a risk-minimizing strategy and that the scheme will offer protection. As noted in the literature, many schemes are poorly managed [6,52,53]. Moreover, in low-capacity environments, quality of care of covered services is often poor and therefore the features of CBHI may not be risk-minimizing relative to other options. For example, individuals who perceive the public health care system to be of low quality may view enrolling in CBHI (which requires users to first seek services at the district hospital in the public system, where quality is reportedly poor) as less attractive than remaining uninsured. Thus, purchasing insurance for some could actually be considered more risky than not purchasing insurance, especially among the poor, for whom a given loss can be ruinous [54-56]. The findings on the relationship between risk and enrolment in CBHI in this study are consistent with a study that found that risk-averse households are less likely to purchase rainfall insurance among farmers in India [57].

Finally, the study findings indicate that the perception of quality of care is an important factor affecting enrolment in CBHI in that those who perceive quality to be poor are less likely to enrol. The complaints about low quality of public health care in Laos are consistent with what other studies from Laos have reported [18,21]. Poor quality of care remains a source of dissatisfaction for *both* members and non-members and is one of the major reasons for leaving the scheme. A recently published study from Laos confirms that cash-paying patients receive more expensive drugs than insured patients but that the insured receive more appropriate care than the uninsured as a result [58]. However, based on the qualitative findings, it seems that the insured perceive themselves as being underserviced in terms of the number of drugs received: in the FGDs, even non-members claim that treatment is better for paying patients. Differential treatment would not be surprising given that capitation payments are low and do not cover costs, and that providers depend on the fees charged by paying patients to operate the facilities. Thus, strategies that aim to improve quality overall, and improve equality of treatment between the insured and uninsured will be important factors affecting insurance uptake in the future. If people do not perceive the health services as valuable, they will be less inclined to enrol. In a review of lessons learned from CBHI schemes in sub-Saharan Africa, Wiesman and Jütting state that quality improvement should not be expected as an outcome of resource mobilisation via insurance, but must be considered a necessary precondition for successful implementation of CBHI [59]. Another study in China suggests that one strategy for promoting better quality, while containing costs, is to delink income of health facilities from drug sales [60]. Improving quality of care in Lao PDR will require greater government investment, for example, in facilities and equipment, human resources, or increased financing of recurrent costs.

The second research question explored in this study focused on the likelihood that CBHI can be expanded further in Laos by comparing the profiles of districts with and without CBHI. The district level findings show that people in districts where CBHI is operating reside closer to the health facilities, and that these districts are more densely populated, more urban, have lower poverty rates, higher literacy rates, and a lower percentage of ethnic minorities. However, even though it is expected that these districts would be the “easiest to reach”, CBHI still only serves a fraction of the target population after more than a decade since the inception of the scheme. As the MOH attempts to expand the scheme to new areas - that are *further* away from health facilities, *less* densely populated, *more* rural, and with *higher* poverty rates, *lower* literacy rates, and a *higher* percentage of ethnic minorities - recruiting new members will likely prove even more difficult.

The study has shown that CBHI in Laos is not adequately pooling risks across the healthy, sick, rich, and poor. The low enrolment, combined with adverse selection, threatens countries’ ability to raise sufficient revenues and achieve financial sustainability. After more than a decade of reaching the most accessible areas, the scheme still only reaches a small fraction of the target population. Global experience has shown that CBHI and other voluntary schemes have failed to achieve high coverage rates [5,10,61,62]. Yet CBHI remains a cornerstone of the Government’s plan for achieving universal coverage and is the only scheme that currently targets the large informal sector. Given this, what are the options for building on the experiences to date to make broad-based progress towards universal coverage?

While some countries have attempted to rely on voluntary schemes to cover the informal sector, more successful reforms have extended tax-financed, or subsidized mandatory schemes to the informal sector. Thailand, for example, experimented with a CBHI scheme for several decades but after evidence showed that the voluntary scheme was not achieving high coverage rates in the informal sector, the scheme was eventually rolled into a tax-financed scheme, which covers the poor and non-poor informal sector and all those not covered by the formal sector schemes. Thailand’s new (mandatory) tax-financed scheme, known as the Universal Coverage scheme, has proven to be a much more effective, equitable and efficient means of covering the informal sector and administratively, is much less complex [63,64]. Mexico has also been successful in expanding insurance by offering households not covered by the formal sector, the option of enrolling in a separate subsidized public health insurance program known as *Seguro Popular*. The contribution is fully subsidized for the poor, while the non-poor make a contribution according to their ability to pay [65]. In contrast, countries such as Vietnam, the Philippines, and Colombia have attempted to cover the informal sector through extension of SHI, but the success of doing so depends on the formalization of the labor market and effectiveness of enforcement mechanisms. In environments where enforcement and regulatory capacity are weak, and the formal sector is small, covering the informal sector through a combination of taxation and donor funds could be administratively more efficient than trying to expand or mandate enrolment among the informal sector. Thus, a valuable lesson from these countries is that social protection works best when it is not tied to employment status.

It is also clear that a key requirement for achieving universal coverage is an increase in government funding [66]. It is true that large informal economies make automatic payroll or tax deductions difficult to implement or enforce on a broad scale, but increasing government revenues is generally feasible even in the poorest countries. A recent review of nine countries that have made progress toward universal coverage found that all countries have increased government spending as a percentage of total health expenditure since launching the reforms (between 5 and 11 percentage points in Ghana, Rwanda, Vietnam, and Indonesia, and between 1 and 3 percentage points in India, Kenya, Mali, and Nigeria). In India, Indonesia, Ghana, Nigeria, Vietnam, and the Philippines, the government increased tax revenues to fund coverage expansion, despite the challenges of tax collection. In other countries, increases in revenues have come from a mix of prepayment mechanisms that, in addition to general taxation, include earmarked taxes, value-added taxes, payroll deductions, and (to a lesser extent) household premium contributions. By increasing revenues, most countries have reduced out-of-pocket household payments for health care and have extended subsidies to target populations such as the poor, pregnant women, and children [67]. It is this combination of a rise in health spending per capita, and a rising share of pooled health expenditures within this total, that characterize the health financing transition [66].

Although government spending on health in Laos is very low by international standards, there are good prospects for government spending on health to increase over the coming years: hydropower and other natural resource revenues have gained importance, and improved tax administration is contributing to increased revenue collection. Both these factors will help generate increased fiscal space over the medium term, and provide an opportunity to significantly increase government health spending. But where should increased government spending be directed? There are a number of options. Government could increase investment spending to improve facilities and equipment; it could invest in human resources for longer-term improvement of service quality; it could increase financing of non-wage recurrent costs at facility level, and in that way reduce reliance on out-of-pocket payments (at least for some priority services); and it could provide direct subsidies to CBHI and Health Equity Funds to increase enrollment and expand benefits of these schemes. Alternatively, it may be feasible to finance a limited package of essential services for those not covered by formal insurance schemes. In fact, the government is currently piloting a project that provides free deliveries in facilities. Finally, alongside these investments, cost-containment and measures to increase efficiencies in the health system will need to be considered.

Recognizing the limitations of CBHI, it is important to consider alternative or complementary financing mechanisms, such as taxation or extension of social health insurance. These are by no means easy options: universal tax-financed schemes require adequate and sustained government financing; targeted schemes require effective targeting mechanisms; expansion of social security depends on increased formalization of the labor market and effective enforcement mechanisms; and contributory schemes require effective and efficient collection systems. Moreover, regardless of the financing approach, mechanisms for promoting quality and efficiency in service delivery are needed. That the Government of Lao PDR has clearly articulated a vision for achieving universal coverage is a good start. However, it is clear from implementation and enrolment to date that the current path to universal coverage will be an uphill battle. In order to achieve broad coverage of key health services and improve financial protection, it will be important to continue revising the health financing strategy, using both evidence from health systems reform within Laos and experience from other countries.

## Conclusions

As low- and middle-income countries make the transition to universal coverage, CBHI and other voluntary schemes are often introduced with the objective of covering the informal sector. Evidence from this study and others shows that voluntary schemes often achieve poor risk-pooling that pose a threat to the schemes’ financial sustainability. In this study, household-level findings indicate that the better-off, less healthy, and less risk-averse are most likely to enroll in insurance, and that poor quality of care prevents households from enrolling in health insurance. The district-level findings call into question whether or not the Government of Laos can successfully expand to more remote, less affluent districts, with lower population density. Thus, the evidence suggests that countries attempting to increase coverage through voluntary health insurance will face difficulties. Although there is no blueprint for achieving universal coverage, it is clear that delinking employment status and coverage is important, and that increased government investment is a prerequisite for universal coverage.

## Abbreviations

CBHI: Community-based health insurance; FGD: Focus group discussion; HEF: Health equity fund; Lao PDR: Lao Peoples’ Democratic Republic; LSHTM: London School of Hygiene and Tropical Medicine; MOH: Ministry of Health; OOP: Out-of-pocket payments; SASS: State authority for social security; SSO: Social Security Organization; UHC: Universal health coverage; VHI: Voluntary health insurance; WHO: World Health Organization.

## Competing interests

The authors have reviewed the competing interests questions and declare that there are no competing financial interests.

## Authors’ contributions

SA participated in the study design, drafted the quantitative and qualitative data collection instruments, supervised data collection, conducted statistical and qualitative analysis, and was the lead writer for the manuscript. ML conceptualized the study and contributed to the design, helped develop both quantitative and qualitative data collection instruments, provided detailed advice and feedback at every stage of the study, and helped draft the manuscript. BJ participated in the design and coordination of the study, provided detailed advice and feedback at every stage of the study, helped gather important secondary data needed for the study, and helped draft the manuscript. All authors read and approved the final manuscript.

## Pre-publication history

The pre-publication history for this paper can be accessed here:

http://www.biomedcentral.com/1472-6963/13/521/prepub
